# Dyslipidemia and its associated factors among community adults located in Shangcheng district, Zhejiang province

**DOI:** 10.1038/s41598-024-54953-6

**Published:** 2024-02-21

**Authors:** Mingming Shi, Hui Wang, Xiao Zhang

**Affiliations:** 1 Hangzhou First People’s Hospital, Hangzhou, Zhejiang China; 2 Center for Disease Control and Prevention of Shangcheng District, Hangzhou, Zhejiang China

**Keywords:** Risk factors, Dyslipidaemias

## Abstract

Dyslipidemia is highly prevalent and an important modifiable risk factor of cardiovascular disease in China. However, there is little information on the dyslipidemia in Shangcheng district, eastern China. Therefore, this study aims to investigate the prevalence and associated factors of dyslipidemia among community adults in this area. A community based cross-sectional study was conducted from August 1 to November 30, 2020. The study utilized a multi-stage probability sampling method to enroll permanent residents (those who have resided in this region for 6 months or more) who were 18 years old or above. Firstly, five streets were selected randomly, and then two communities were randomly selected from each of the chosen streets, finally, systematic sampling at the household level was conducted. All participants were interviewed by trained investigators and underwent anthropometric and biochemical measurements using standard criteria. LASSO (least absolute shrinkage and selection operator) and multivariate binary logistic regression were employed to identify the factors associated with dyslipidemia. In total, 3153 participants were enrolled into this study, resulting in a response rate of 93.28%. 33 subjects were excluded because of incomplete data. Finally, 3120 participants with a mean age of 55.26 (SD = 17.97) years were included into analysis. The overall prevalence of dyslipidemia was 35.96%. 21 variables were screened to multivariate binary logistic regression through the implementation of LASSO method. The multivariate binary logistic regression analysis revealed that individuals aged 40–49 [adjusted odds ratio (aOR) = 2.197, 95% confidence interval (CI) 1.445–3.341], 50–59 (aOR = 3.213, 95% CI 2.121–4.868), 60–69 (aOR = 4.777, 95% CI 3.169–7.201), and 70 and above (aOR = 5.067, 95% CI 3.301–7.777), with an educational level of junior middle school (aOR = 1.503, 95% CI 1.013–2.229), with an educational level of senior middle school (aOR = 1.731, 95% CI 1.25–2.397), with an educational level of under graduate and above (aOR = 2.125, 95% CI 1.46–3.095), without hypertension (aOR = 0.627, 95% CI 0.517–0.76), without diabetes (aOR = 0.625, 95% CI 0.498–0.785), obesity (aOR = 1.887, 95% CI 1.13–3.154), frequent smoking (aOR = 1.727, 95% CI 1.293–2.308), frequent drinking (aOR = 0.738, 95% CI 0.556–0.981), without family history of CVD (aOR = 0.505, 95% CI 0.342–0.744), and daily seafood intakes between 42.87 and 71.43 g (aOR = 1.31, 95% CI 1.05–1.634) were significantly associated with dyslipidemia. Gender-stratified analyses showed that aged 70 and above (aOR = 2.127, 95% CI 1.195–3.785), without hypertension (aOR = 0.643, 95% CI 0.484–0.854), without diabetes (aOR = 0.603, 95% CI 0.436–0.834), without CVD (aOR = 0.494, 95% CI 0.309–0.791), without stroke (aOR = 1.767, 95% CI 1.036–3.012), frequent smoking (aOR = 1.951, 95% CI 1.415–2.691), former smoking (aOR = 1.703, 95% CI 1.16–2.502) were significantly associated with dyslipidemia in male. Aged 40–49 (aOR = 3.51, 95% CI 1.789–6.887), 50–59 (aOR = 7.03, 95% CI 3.584–13.791), 60–69 (aOR = 15.728, 95% CI 8.005–30.9), and 70 and above (aOR = 12.929, 95% CI 6.449–25.921), with an educational level of senior middle school (aOR = 1.926, 95% CI 1.288–2.881), with an educational level of under graduate and above (aOR = 2.91, 95% CI 1.75–4.837), without hypertension (aOR = 0.592, 95% CI 0.45–0.779), without diabetes (aOR = 0.619, 95% CI 0.443–0.865), without family history of CVD (aOR = 0.429, 95% CI 0.251–0.733), without family history of cancer (aOR = 0.542, 95% CI 0.316–0.929), daily vegetables intakes between 251 and 500 g (aOR = 0.734, 95% CI 0.545–0.99), daily seafood intakes between 42.87 and 71.43 g (aOR = 1.421, 95% CI 1.04–1.942) were significantly associated with dyslipidemia in female. In the age-stratified analyses, it was found that without hypertension (aOR = 0.522, 95% CI 0.375–0.727) or diabetes (aOR = 0.445, 95% CI 0.267–0.744), obesity (aOR = 2.956, 95% CI 1.258–6.942), frequent smoking (aOR = 1.826, 95% CI 1.196–2.787), showed a significant association with dyslipidemia in individuals aged younger than 60 years. Female (aOR = 1.764, 95% CI 1.316–2.366), with an educational level of junior middle school (aOR = 1.793, 95% CI 1.169–2.749), with an educational level of senior middle school (aOR = 2.002, 95% CI 1.406–2.849), with an educational level of under graduate and above (aOR = 2.849, 95% CI 1.791–4.532), without hypertension (aOR = 0.604, 95% CI 0.477–0.764), without diabetes (aOR = 0.63, 95% CI 0.486–0.818), without CVD (aOR = 0.66, 95% CI 0.473–0.921), frequent smoking (aOR = 1.513, 95% CI 1.02–2.245), former smoking (aOR = 1.647, 95% CI 1.089–2.491), without family history of CVD (aOR = 0.406, 95% CI 0.239–0.692), daily seafood intakes between 42.87 and 71.43 g (aOR = 1.376, 95% CI 1.018–1.859) were significantly associated with dyslipidemia among participants aged 60 and above. Dyslipidemia is a prevalent condition observed among adults residing in Shangcheng district. Risk factors such as gender, age, education, hypertension, diabetes, cardiovascular disease, stroke, obesity, smoking, drinking, family history of cardiovascular disease, family history of cancer, daily vegetables intakes, daily seafood intakes were associated with dyslipidemia and varied across population of different gender and age groups. Enhancing education and promoting self-awareness regarding the necessity of behavior modification and regular medication intake would be beneficial in reducing the occurrence of dyslipidemia among adults in the Shangcheng district.

## Introduction

Cardiovascular disease (CVD) is a leading cause of death worldwide, accounting for 31% mortality globally in 2016^1^. Compared to the significant decline in cardiovascular disease (CVD) mortality observed in developed western nations^2^, China is currently facing a notable rise in the incidence and death rates associated with CVD, coinciding with rapid societal and economic development. Research indicated that the estimated cases of cardiovascular disease was 93.8 million in 2016, which is more than twice those in 1990. Moreover, there was an increase in cardiovascular disease-related mortality from 2.51 million to 3.97 million between 1990 and 2016^3^.

Dyslipidemia is one of the most indisputable risk factors for developing and progressing the atherosclerosis^4^, it is modifiable by medication and lifestyle changes. For CVD patients, it is generally agreed upon by guideline that serum lipid control is necessary. Dyslipidemia is a metabolism disorder characterized by any or a combination of the following: elevated blood levels of total cholesterol (TC), elevated triglycerides (TG), elevated low-density lipoprotein cholesterol (LDL-C), and reduced concentrations of high-density lipoprotein cholesterol (HDL-C). The prevalence of dyslipidemia among adults in China experienced a significant rise from 18.60% in 2002 to 40.40% in 2012, indicating a increase of 21.8 percent over the decade^5,6^. The prevalence of dyslipidemia in China is notably higher than that of other Asian countries with similar economies, such as the Republic of Korea and Japan^7,8^. Managing population lipid levels has garnered increased attention, as the optimal management of dyslipidemia is crucial for the prevention of cardiovascular disease (CVD)^9^.In July 2019, the Chinese government introduced the "Healthy China Action (2019–2030)", which encompasses 'Action against cardiovascular disease' as one of the fifteen key initiatives, with a specific emphasis on expanding lipid testing across the nation^10^.

Shangcheng district, located in the capital city of Hangzhou in Zhejiang province, is characterized by a stable population with a significant aging problem. The district has been designated as one of the initial national chronic disease prevention and control demonstration areas as well as the first demonstration site for blood lipid prevention and control in Hangzhou. However, there is scarce epidemiological data on the prevalence of dyslipidemia among adults in this area. Due to the significant variation in diets and lifestyles across China^11^, it is not feasible to extrapolate directly from data obtained in other regions. While a number of studies have examined the epidemiology of dyslipidemia in Zhejiang province, these studies were constrained by specific populations or insufficient estimation^12–14^. On the other hand, the epidemiology of dyslipidemia is dynamic phase, precise ongoing data about lipid status in community population had more practical value. In light of these circumstances, we intend to conduct a community-based cross-sectional study to assess the burden of dyslipidemia among community adults in Shangcheng district, and to identify potential associated factors.

## Method

### Study population

The sample size was estimated based on the following formula:

$$N = deff\frac{{u^{2} p(1 - p)}}{{(pd)^{2} }}$$

The confidence interval (u) used in this study is set at 95%, with the corresponding value of u = 1.96. The rate of disease (p) in this context refers to the prevalence of diabetes mellitus in Zhejiang Province, which is reported to be 7.37%^6^. The design efficiency (deff) for the present study is set at a value of 1.4. The error tolerance (d) is set at 20%. To account for potential non-response, an additional 10% was added to the minimum sample size. The gender-ratio of individuals aged 18 years and older data for the Shangcheng district in 2020, provided by public security authorities, was used to calculate the sample size, the sample size calculated to be 3380. A multi-stage sampling method was utilized, first, five streets were chosen randomly, second, two communities were randomly selected from each of the chosen streets. Resident registration information was obtained from the selected Community residents' committee, and systematic sampling at the household level was conducted. Survey participants included all family members over 18 years in the sampled households. The inclusion criteria for the study participant were: (a) aged 18 years old and above; (b) living in the selected areas 6 months and above. The exclusion criteria were: (a) physical disability; (b) living in the selected areas less than 6 months and (c) psychological or mental health issues.

Ethical approval was obtained from Academic Review Board (ARB) of Center for Disease Control and Prevention of Shangcheng District, (approval number 202007, dated June 2020). Written informed consent was obtained from the participant before the survey.

### Questionnaire investigation

Study was conducted from August 1 to November 30, 2020. Trained interviewers conducted a face-to-face interview at a local community health center using structured questionnaires. The questionnaires covered socio-demographic characteristics, health-related information, lifestyle information, and the frequency and consumption of various food items. Information regarding dietary consumption was collected through food frequency questionnaire (FFQ) that is widely employed in mainland China^15,16^. Participants were required to report their frequency of consumption of various food weekly over the past year, as well as the quantity of each food item consumed during each instance. All completed questionnaires were subjected to review by supervisors and entered into the system using a parallel double-entry method.

### Anthropometric measurements

Trained investigators performed the physical examination, the anthropometric measurements of each participants were performed using a standardized protocol. Height, weight and waist circumference were measured with participants wearing light indoor clothing and no shoes. Participants' weight was measure in kilogram(kg) to the nearest 0.1 kg and height was measured to the nearest 0.1 cm using statiometer, Participants' waist circumference was measured to the nearest 0.1 cm using waist measuring tape. BMI was calculated as an individual's weight in kilograms divided by height in meters squared. A standardized automatic electronic sphygmomanometer was used to measure blood pressure of the participants. A resting period of at least 5 min was required before measurement was taken. The measurement was performed three times and the average value was used in the analysis.

### Biochemical measurement

The blood samples of 10 ml were taken from the antecubital vein will be obtained after an overnight fast(≥ 10 h). The blood samples clotted and centrifuged, then freezed and stored below − 20 °C and send to local medical center laboratory for testing immediately. The level of glucose, TC, TG, HDL-C, and LDL-C were measured. All laboratory measurements were done as per guideline. The standardized procedures were strictly followed during the blood sample collection, storage and analysis. The entire process is done by trained medical staff.

### Definition of variables

Dyslipidemia: total cholesterol (TC) ≥ 6.2 mmol/L and/or triglyceride (TG) ≥ 2.3 mmol/L and/or high-density lipoprotein cholesterol (HDL-C) ≤ 1.0 mmol/L and/or low-density lipoprotein cholesterol (LDL-C) ≥ 4.1 mmol/L or currently using lipid-lowering agents, or previously diagnosed with dyslipidemia by a doctor^17^ .

Diabetes: FBG ≥ 7.0 mmol/L, oral glucose tolerance test (OGTT-2 h) ≥ 11.0 mmol/L plasma glucose or self-reported use of anti-diabetic medication in the 2 weeks prior to the examination or self-reported prior diagnosis of diabetes by a doctor^18^.

Hypertension was defined as systolic pressure > 140 mmHg or diastolic pressure > 90 mmHg or self-reported current use of antihypertensive medication, or prior diagnosis of hypertension by a doctor^19^.

Body mass index (BMI) was calculated by dividing the weight by the square of the height(kg/m^2^), obesity status was defined in accordance with the obesity standards in China (< 18.5 kg/m^2^ for underweight, 18.5–23.9 kg/m^2^ for normal, 24–27.9 kg/m^2^ for overweight, ≥ 28 kg/m^2^ for obese)^20^.

Waist circumference (WC) was measured at the midpoint between the iliac crest and the lower rib. Men with waist circumference ≥ 90 cm or women with waist circumference ≥ 85 cm were defined as high^20^.

In present study, the smoking status is categorized into four distinct groups: never, occasional, frequent and former. The term ‘frequent’ denotes individuals who have smoked at least one cigarette per day for a period of six months or more, and are presently continuing to smoke. Meanwhile, ‘occasional’ smokers are those who still engage in smoking but do not consume at least one cigarette per day^21^.

Secondhand smoke is the inhalation of smoke by individuals who do not smoke cigarettes^21^.

In the current study, the consumption of alcohol is categorized into four groups: never, occasional, frequent and former. The term ‘frequent’ denotes individuals who consume alcohol at least once a week and continue to drink. ‘occasional’ drinker is one who still consumes alcohol but does less than once per day^22^.

A history of CVD, stroke and cancer was defined as self-report of any previous diagnosis of CVD, stroke and cancer by a healthcare professional or currently undergoing treatment.

A family history of CVD, hypertension, diabetes, stroke and cancer was defined as a positive response on the survey to questions regarding their parents or siblings being diagnosed with disease mentioned above^23^.

Dietary information: participants were requested to remember how often they consumed different food items on a weekly and the quantity of each item. These responses were then converted into daily intake amounts. The food categories primarily consisted of grains, vegetables, fruits, meat, seafood, eggs, and dairy products. We utilized the quartile method for categorization because of the non-normal distribution of the dietary data.

### Statistics

Data analysis was performed using SPSS software (version 23.0) and R statistical software (version 3.6.2). Data were divided into two groups based on the presence or absence of dyslipidemia. Continuous variables are expressed as either mean ± standard deviation (SD) or median(first quartile-third quartile). The Student’s t-test is employed to assess variables that adhere to a normal distribution, while the Mann–Whitney U test is utilized for variables that do not adhere to a normal distribution. Categorical variables were represented as frequencies and percentages, and the chi-squared test was employed to compare disparities between groups. We utilized the LASSO (Least absolute shrinkage and selection operator) method, a modern and reliable statistical technique, to screen factors associated with dyslipidemia. Ten-fold cross-validation was used to select the penalty term λ. The λ value corresponding to the minimum binomial deviance was chosen, and factors were filtered. The LASSO analysis was performed using the "glmnet" package with R software. The entire variables selected through LASSO were incorporated into multivariate binary logistic regression analyses. Additionally, stratified analyses by gender and age were analyzed, with age groups categorized based on a 60 year cut-off, due to the sample sizes after grouping. P < 0.05 was considered statistically significant.

## Results

**Figure 1:**
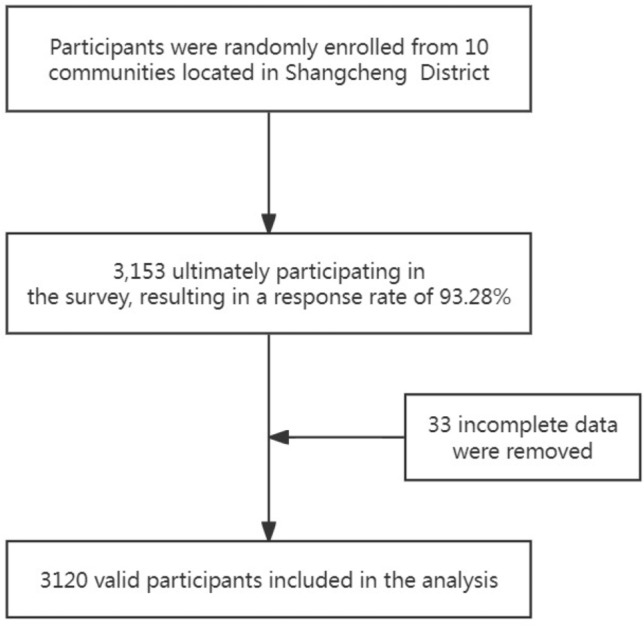
Shows the flow chart of population selection.

3153 participants were enrolled into present study, with response rate of 93.28% (3153/3380). 33 were excluded due to incomplete data. Finally, 3120 participants aged from 18 to 95 were included in the analysis, the flowchart of population selection was shown in Fig. 1. The general characteristics of the participants are presented in Table 1. The dyslipidemia group and the non-dyslipidemia group had a similar pattern of demographic characteristics with a majority of those not secondhand smoker, without stroke, without cancer, without family history of diabetes or family history of stroke. Participants with dyslipidemia were more likely to have hypertension, diabetes, obesity .

**Table 1:** Demographic characteristics of the participants.

Variable	Total (n = 3120)	With dyslipidemia (n = 1122)	Without dyslipidemia (n = 1998)	*x*^2^/Z	*p*
Age, n (%)				281.9	< 0.01
18–29	302	39(12.9)	263(87.1)		
30–39	481	74(15.4)	407(84.6)		
40–49	369	103(27.9)	266(72.1)		
50–59	479	174(36.3)	305(63.7)		
60–69	765	368(48.1)	397(51.9)		
70 and above	724	364(50.3)	360(49.7)		
Gender				0.85	0.36
Male	1409	519(36.8)	890(63.2)		
Female	1711	603(35.2)	1108(64.8)		
Education, n (%)				67.48	< 0.01
Primary school and below	217	81(37.3)	136(62.7)		
Junior middle school	244	110(45.1)	134(54.9)		
Senior middle school	1588	648(40.8)	940(59.2)		
Under graduate and above	1071	283(26.4)	788(73.6)		
Occupation				178.58	< 0.01
None	1806	826(45.7)	980(54.3)		
Mental labour	1016	223(21.9)	793(78.1)		
Physical labor	17	4(23.5)	13(76.5)		
Unknown	281	69(24.6)	212(75.4)		
Marriage status				88.2	< 0.01
Unmarried	385	59(15.3)	326(84.7)		
Married	2313	879(38)	1434(62)		
Cohabitation	128	51(39.8)	77(60.2)		
Widowed	212	99(46.7)	113(53.3)		
Divorce	73	31(42.5)	42(57.5)		
Separation	9	3(33.3)	6(66.7)		
Hypertension, n (%)				197.97	< 0.01
No	1984	532(26.8)	1452(73.2)		
Yes	1136	590(51.9)	546(48.1)		
Diabetes, n (%)				115.04	< 0.01
No	2663	856(32.1)	1807(67.9)		
Yes	457	266(58.2)	191(41.8)		
CVD, n (%)				42.41	< 0.01
No	2895	996(34.4)	1899(65.6)		
Yes	217	122(56.2)	95(43.8)		
Unknown	8	4(50)	4(50)		
Stroke, n (%)				8.25	0.02
No	2978	1055(35.4)	1923(64.6)		
Yes	137	65(47.4)	72(52.6)		
Unknown	5	2(40)	3(60)		
Cancer, n (%)				4.48	0.11
No	2975	1058(35.6)	1917(64.4)		
Yes	141	62(44)	79(56)		
Unknown	4	2(50)	2(50)		
Waist circumference, n (%)				63.51	< 0.01
Normal	2365	759(32.1)	1606(67.9)		
High	755	363(48.1)	392(51.9)		
BMI, n (%)				79.01	< 0.01
Underweight	155	48(31)	107(69)		
Normal	1864	571(30.6)	1293(69.4)		
Overweight	911	398(43.7)	513(56.3)		
Obesity	190	105(55.3)	85(44.7)		
Exercise				0.69	0.4
No	1173	411(35)	762(65)		
Yes	1947	711(36.5)	1236(63.5)		
Smoke				40.89	< 0.01
Never	2438	813(33.3)	1625(66.7)		
Frequent	400	180(45)	220(55)		
Occasional	93	32(34.4)	61(65.6)		
Former	189	97(51.3)	92(48.7)		
Secondhand smoker, n (%)				1.49	0.22
No	2372	839(35.4)	1533(64.6)		
Yes	748	283(37.8)	465(62.2)		
Drink				20.25	< 0.01
Never	2227	755(33.9)	1472(66.1)		
Frequent	348	142(40.8)	206(59.2)		
Occasional	449	175(39)	274(61)		
Former	96(	50(52.1)	46(47.9)		
Family history of hypertension, n (%)				26.88	< 0.01
No	1942	631(32.5)	1311(67.5)		
Yes	1178	491(41.7)	687(58.3)		
Family history of diabetes, n (%)				4.07	0.04
No	2696	951(35.3)	1745(64.7)		
Yes	424	171(40.3)	253(59.7)		
Family history of CVD, n (%)				34.38	< 0.01
No	2984	1041(34.9)	1943(65.1)		
Yes	136	81(59.6)	55(40.4)		
Family history of stroke, n (%)				2.16	0.14
No	3007	1074(35.7)	1933(64.3)		
Yes	113	48(42.5)	65(57.5)		
Family history of cancer, n (%)				6.53	0.01
No	2969	1053(35.5)	1916(64.5)		
Yes	151	69(45.7)	82(54.3)		
Daily grains intake, n (%)				2.75	0.43
Q1 (≤ 200 g)	1195	409(34.2)	786(65.8)		
Q2 (201–250 g)	656	246(37.5)	410(62.5)		
Q3 (251–300 g)	720	268(37.2)	452(62.8)		
Q4 (≥ 301 g)	549	199(36.2)	350(63.8)		
Daily vegetables intake, n (%)				4.51	0.21
Q1(0–200 g)	858	286(33.3)	572(66.7)		
Q2(201–250 g)	977	350(35.8)	627(64.2)		
Q3(251–500 g)	1150	435(37.8)	715(62.2)		
Q4(501– g)	135	51(37.8)	84(62.2)		
Daily fruits intake, n (%)				2.64	0.45
Q1(≤ 57.14 g)	814	297(36.5)	517(63.5)		
Q2(57.15–100 g)	941	319(33.9)	622(66.1)		
Q3(100.01–150 g)	638	234(36.7)	404(63.3)		
Q4(≥ 150.01 g)	727	272(37.4)	455(62.6)		
Daily meat intake, n (%)				11.99	< 0.01
Q1(≤ 28.57 g)	872	353(40.5)	519(59.5)		
Q2(28.58–57.14 g)	846	296(35)	550(65)		
Q3(57.15–100 g)	831	272(32.7)	559(67.3)		
Q4(≥ 100.01 g)	571	201(35.2)	370(64.8)		
Daily seafood intakes, n (%)				7.08	0.07
Q1(≤ 28.57 g)	1094	373(34.1)	721(65.9)		
Q2(28.58–42.86 g)	607	204(33.6)	403(66.4)		
Q3(42.87–71.43 g)	675	264(39.1)	411(60.9)		
Q4(≥ 71.44 g)	744	281(37.8)	463(62.2)		
Daily soy and soy product intake, n (%)				5.21	0.16
Q1(≤ 14.29 g)	1055	357(33.8)	698(66.2)		
Q2(14.3–28.57 g)	638	225(35.3)	413(64.7)		
Q3(28.58–57.14 g)	722	265(36.7)	457(63.3)		
Q4(≥ 57.15 g)	705	275(39)	430(61)		
Daily milk and milk products intake, n (%)				15.70	< 0.01
Q1(≤ 14.29 g)	794	319(40.2)	475(59.8)		
Q2(14.30–142.86 g)	847	262(30.9)	585(69.1)		
Q3(142.87–227.5 g)	699	254(36.3)	445(63.7)		
Q4(≥ 227.51 g)	780	287(36.8)	493(63.2)		
Daily nuts intake, n (%)				5.34	0.07
Q1(0 g )	1741	604(34.7)	1137(65.3)		
Q2(0.01–7.14 g)	413	142(34.4)	271(65.6)		
Q3(≥ 7.15 g)	966	376(38.9)	590(61.1)		
Daily eggs intake, n (%)				1.45	0.23
≤ 1 egg	2982	1079(36.2)	1903(63.8)		
> 1 egg	138	43(31.2)	95(68.8)		
Sleeping duration, n (%)				32.48	< 0.01
Q1(≤ 6 h)	853	370(43.4)	483(56.6)		
Q2(6.1–7 h)	906	289(31.9)	617(68.1)		
Q3(7.1–8 h)	1057	346(32.7)	711(67.3)		
Q4(≥ 8.1 h)	304	117(38.5)	187(61.5)		
Resting time, n (%)				6.82	0.08
Q1(≤ 2 h)	964	352(36.5)	612(63.5)		
Q2(2.01–4 h)	992	367(37)	625(63)		
Q3(4.01–6 h)	636	239(37.6)	397(62.4)		
Q4(≥ 6 h)	528	164(31.1)	364(68.9)		
TC, mmol/L, median(first quartile–third quartile)	4.78(4.13–5.43)	5.12(4.31–6.24)	4.66(4.07–5.15)	13.42	< 0.01
TG, mmol/L, median(first quartile–third quartile)	1.38(1.02–1.89)	1.88(1.31–2.65)	1.23(0.91–1.57)	24.33	< 0.01
HDL-C, mmol/L, median(first quartile–third quartile)	1.38(1.18–1.66)	1.28(0.99–1.55)	1.43(1.25–1.7)	-14.23	< 0.01
LDL-C, mmol/L, median(first quartile–third quartile)	2.64(2.12–3.16)	2.87(2.23–3.48)	2.56(2.09–3.01)	10.68	< 0.01

*CVD* cardiovascular disease, *BMI* body mass index, *TG* triglyceride, *TC* serum total cholesterol, *HDL* high-density lipoprotein, *LDL* low-density lipoprotein;

Table 2 shows the lipid levels in the subgroups by gender and age. Male had higher levels of TG than female, while female had higher level of TC, HDL-C and LDL-C than male. Compared to the 18–29 years group, the level of TC, TG and LDL-C increased with age and were highest in the 50–59 year. HDL-C tended to decrease with age.

**Table 2:** Median (IQR) of serum TC, TG, HDL-C, LDL-C among community adults in Shangcheng District.

Categories	TC(mmol/L)	TG(mmol/L)	HDL-C(mmol/L)	LDL-C(mmol/L)
Total	4.78(4.13–5.43)	1.38(1.02–1.89)	1.38(1.18–1.66)	2.64(2.12–3.16)
Gender-specific				
Male	4.7(4.05–5.26)	1.42(1.05–1.94)	1.32(1.11–1.56)	2.63(2.11–3.14)
Female	4.84(4.19–5.56)	1.35(0.98–1.84)	1.44(1.24–1.71)	2.66(2.15–3.18)
P	< 0.01	< 0.01	< 0.01	0.12
Age-specific, (year)				
18–29	4.51(3.89–5.02)	1.21(0.87–1.59)	1.51(1.29–1.83)	2.53(2.05–3.07)
30–39	4.6(3.97–5.1)	1.24(0.92–1.59)	1.43(1.23–1.69)	2.68(2.18–3.09)
40–49	4.75(4.1–5.32)	1.46(1.02–2.04)	1.36(1.18–1.61)	2.57(2.19–3.11)
50–59	4.98(4.37–5.61)	1.45(1.07–1.98)	1.4(1.2–1.69)	2.76(2.22–3.22)
60–69	4.88(4.28–5.67)	1.48(1.1–1.99)	1.35(1.14–1.61)	2.68(2.14–3.21)
70 and above	4.8(4.03–5.55)	1.38(1.04–1.9)	1.33(1.12–1.6)	2.58(2.01–3.21)
P	< 0.01	< 0.01	< 0.01	< 0.01
P_trend_	< 0.01	< 0.01	< 0.01	< 0.01
Gender- and age-specific				
Women, age(year)				
18–29	4.39(3.87–4.99)	1.05(0.79–1.44)	1.64(1.35–1.9)	2.44(1.95–2.97)
30–39	4.56(3.9–5.04)	1.12(0.85–1.56)	1.46(1.26–1.73)	2.57(2.12–3.01)
40–49	4.72(4.09–5.28)	1.35(0.92–1.75)	1.39(1.22–1.69)	2.56(2.19–3.04)
50–59	5.06(4.4–5.76)	1.35(1.01–1.89)	1.48(1.26–1.74)	2.73(2.22–3.22)
60–69	5.1(4.38–5.85)	1.51(1.13–2.12)	1.41(1.21–1.64)	2.79(2.2–3.36)
70 and above	5(4.26–5.7)	1.45(1.12–1.97)	1.4(1.19–1.66)	2.74(2.12–3.29)
P	< 0.01	< 0.01	< 0.01	< 0.01
P_trend_	< 0.01	< 0.01	< 0.01	< 0.01
Men, age(year)				
18–29	4.69(4.08–5.1)	1.41(1.04–1.87)	1.38(1.21–1.65)	2.63(2.19–3.17)
30–39	4.7(4.05–5.12)	1.36(1.02–1.69)	1.37(1.16–1.63)	2.84(2.26–3.18)
40–49	4.78(4.19–5.38)	1.76(1.25–2.24)	1.32(1.13–1.55)	2.63(2.16–3.2)
50–59	4.91(4.21–5.47)	1.64(1.25–2.21)	1.34(1.12–1.6)	2.83(2.18–3.21)
60–69	4.68(4.16–5.25)	1.45(1.06–1.9)	1.29(1.08–1.52)	2.57(2.07–3.09)
70 and above	4.58(3.83–5.27)	1.3(0.98–1.82)	1.28(1.05–1.52)	2.41(1.89–3.04)
P	0.01	< 0.01	< 0.01	0.01
P_trend_	0.17	0.29	< 0.01	0.02

Data present as median(first quartile- third quartile).

*TG* triglyceride, *TC* serum total cholesterol, *HDL* high-density lipoprotein, *LDL* low-density lipoprotein.

The prevalence of various types of dyslipidemia is presented in Table 3. The overall prevalence of dyslipidemia was 35.96%. It demonstrated an increasing trend with age (P_trend_ < 0.001), which is also observed in low HDL-C measurement (P_trend_ < 0.001), The prevalence of high TC, high TG and high LDL-C initially rose, and the declined with age(P_trend_ < 0.001). There was no statistically significant difference in the overall prevalence of dyslipidemia between male and female (P = 0.356), but high TC and high LDL-C were significantly higher in female than male, while low HDL-C was significantly higher in male than female.

**Table 3:** The age and gender specific prevalence of different types of dyslipidemia.

Categories	H-TC,n (%)	H-TG,n (%)	L-HDL,n (%)	H-LDL,n (%)	Dyslipidemia,n (%)
Total	556(17.82)	401(12.85)	342(10.96)	122(3.91)	1122(35.96)
Gender-specific					
Male	274(19.4)	134(9.5)	177(12.6)	42(3)	519(36.8)
Female	282(16.5)	267(15.6)	165(9.6)	80(4.7)	603(35.2)
P	0.031	< 0.001	< 0.001	0.015	0.356
Age-specific, (year)					
18–29	21(7)	12(4)	8(2.6)	5(1.7)	39(12.9)
30–39	34(7.1)	21(4.4)	32(6.7)	7(1.5)	74(15.4)
40–49	58(15.7)	33(8.9)	30(8.1)	16(4.3)	103(27.9)
50–59	99(20.7)	65(13.6)	37(7.7)	18(3.8)	174(36.3)
60–69	181(23.7)	143(18.7)	110(14.4)	40(5.2)	368(48.1)
70 and above	163(22.5)	127(17.5)	125(17.3)	36(5)	364(50.3)
P	< 0.001	< 0.001	< 0.001	< 0.003	< 0.001
P_trend_	< 0.001	< 0.001	< 0.001	< 0.001	< 0.001

*H-TC* high total cholesterol, *H-TG* high triglyceride, *H-LDL* high low-density lipoprotein cholesterol, *L-HDL* low high-density lipoprotein cholesterol.

**Figure 2:**
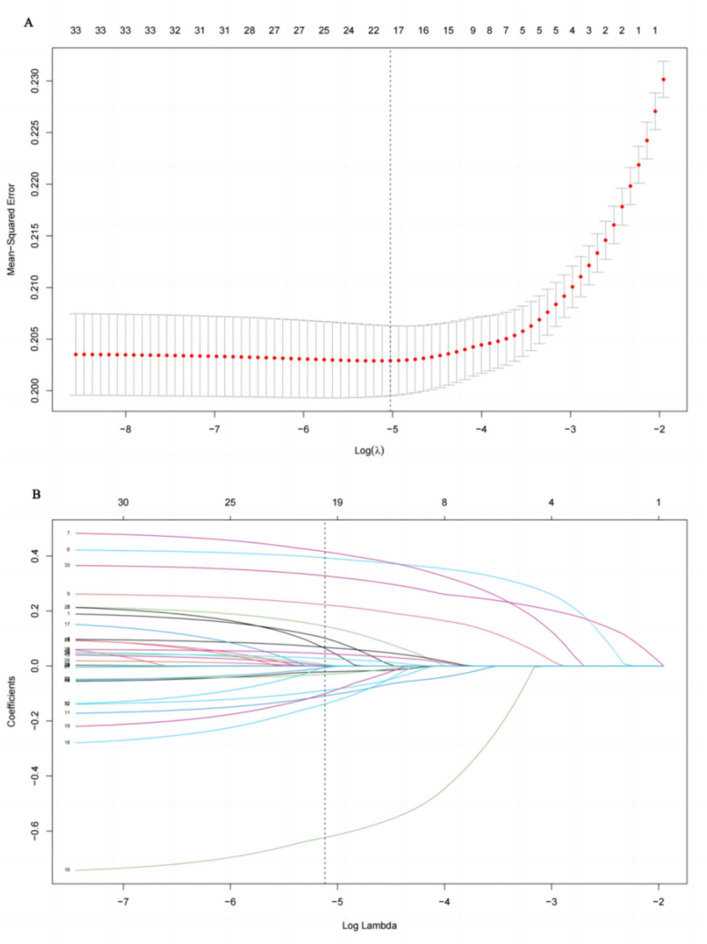
Selection of significant parameters in variables associated with dyslipidemia. (**A**) Ten time cross-validation for tuning parameter selection in the LASSO model. (**B**) LASSO coefficient profiles. The LASSO was used for regression of high dimensional predictors. The method uses an L1 penalty to shrink some regression coefficients to exactly zero. The binomial deviance curve was plotted versus log (λ), where λ is the tuning parameter (**A**). LASSO coefficient profiles of variables (**B**). *LASSO* least absolute shrinkage and selection operator.

Table 4 displays the data for variables related to dyslipidemia identified through LASSO regression. The model achieved minimum binomial deviance when λ = 0.006544998, 21 variables remain in the model (i.e. are non-zero) (Fig. 2). The LASSO regression method was used to identify potential risk factors for dyslipidemia, including age, gender, education, hypertension, diabetes, cardiovascular disease, stroke, waist circumference, BMI, smoking, secondhand smoker, drinking, family history of hypertension, cardiovascular disease, and cancer, daily intake of grains, vegetables, fruits, seafood, milk and dairy products and eggs.

**Table 4:** The estimated coefficients for LASSO regression between associated factors with dyslipidemia.

Variables	Coefficients
Age	0.064
Gender	0.02
Education	0.031
Occupation	
Marriage status	
Hypertension	0.095
Diabetes	0.101
CVD	−0.029
Stroke	0.013
Cancer	
Waist circumference	0.018
BMI	0.045
Exercise	
Smoke	−0.022
Secondhand smoker	−0.015
Drink	0.001
Family history of hypertension	−0.002
Family history of diabetes	
Family history of CVD	−0.137
Family history of stroke	
Family history of cancer	−0.028
Daily grains intake	−0.006
Daily vegetables intake	−0.001
Daily fruits intake	0.006
Daily meat intake	
Daily seafood intakes	0.009
Daily soy and soy product intake	
Daily milk and milk products intake	0.001
Daily nuts intake	
Daily eggs intake	−0.001
Sleeping duration	
Resting time	

*CVD* cardiovascular disease, *BMI* body mass index.

The associated factors of dyslipidemia are shown in Table 5. Results of multivariate binary logistic regression reveals that age, education, hypertension, diabetes, obesity, smoking, drinking, family history of CVD and daily seafood intakes were associated with dyslipidemia.

**Table 5:** The multivariate binary logistic regression model analysis between dyslipidemia and related factors among community adults in Shangcheng district.

Variables	B	Se	Wald *x*^2^	P	OR	Lower	Upper
Gender (ref: male)	0.211	0.11	3.689	0.055	1.234	0.996	1.531
Age							
18–29	1						
30–39	0.092	0.217	0.18	0.671	1.096	0.717	1.676
40–49	0.787	0.214	13.54	< 0.001	2.197	1.445	3.341
50–59	1.167	0.212	30.324	< 0.001	3.213	2.121	4.868
60–69	1.564	0.209	55.76	< 0.001	4.777	3.169	7.201
70 and above	1.623	0.219	55.123	< 0.001	5.067	3.301	7.777
Education							
Primary school and below	1						
Junior middle school	0.407	0.201	4.095	0.043	1.503	1.013	2.229
Senior middle school	0.549	0.166	10.9	0.001	1.731	1.25	2.397
Under graduate and above	0.754	0.192	15.476	< 0.001	2.125	1.46	3.095
Hypertension(ref: yes)	−0.467	0.098	22.601	< 0.001	0.627	0.517	0.76
Diabetes(ref: yes)	−0.469	0.116	16.325	< 0.001	0.625	0.498	0.785
CVD(ref: yes)	−0.293	0.164	3.179	0.075	0.746	0.541	1.03
Stroke(ref: yes)	0.303	0.199	2.329	0.127	1.354	0.917	1.998
WC(ref:normal)	0.089	0.111	0.633	0.426	1.093	0.878	1.359
BMI							
Underweight	1						
Normal	−0.058	0.194	0.089	0.766	0.944	0.645	1.328
Overweight	0.228	0.208	1.205	0.272	1.256	0.836	1.888
Obesity	0.635	0.262	5.88	0.015	1.887	1.13	3.154
Smoke							
Never	1						
Frequent	0.546	0.148	13.663	< 0.001	1.727	1.293	2.308
Occasional	0.024	0.255	0.009	0.924	1.025	0.621	1.69
Former	0.337	0.184	3.337	0.068	1.401	0.976	2.011
Secondhand smoker (ref: yes)	−0.137	0.103	1.777	0.183	0.872	0.713	1.067
Drink							
Never	1						
Frequent	−0.303	0.145	4.375	0.036	0.738	0.556	0.981
Occasional	0.111	0.123	0.803	0.37	1.117	0.877	1.423
Former	0.077	0.231	0.109	0.741	1.08	0.686	1.699
Family history of hypertension (ref: yes)	0.002	0.088	0.001	0.982	1.002	0.843	1.191
Family history of CVD(ref: yes)	−0.683	0.198	11.92	0.001	0.505	0.342	0.744
Family history of cancer(ref: yes)	−0.259	0.183	1.992	0.158	0.772	0.539	1.106
Daily grains intake							
Q1(≤ 200 g)	1						
Q2(201–250 g)	−0.07	0.113	0.387	0.543	0.932	0.746	1.164
Q3(251–300 g)	−0.1	0.112	0.792	0.373	0.905	0.726	1.128
Q4(≥ 301 g)	−0.165	0.13	1.611	0.204	0.848	0.657	1.094
Daily vegetables intake							
Q1(≤ 200 g)	1						
Q2(201–250 g)	−0.103	0.11	0.878	0.349	0.902	0.727	1.119
Q3(251–500 g)	−0.099	0.108	0.849	0.357	0.905	0.733	1.118
Q4(≥ 501 g)	−0.271	0.212	1.629	0.202	0.763	0.503	1.156
Daily fruits intake							
Q1(≤ 57.14 g)	1						
Q2(57.15–100 g)	−0.025	0.113	0.048	0.827	0.976	0.781	1.218
Q3(100.01–150 g)	0.099	0.126	0.621	0.431	1.104	0.863	1.413
Q4(≥ 150.01 g)	0.131	0.124	0.115	0.291	1.14	0.894	1.454
Daily seafood intakes							
≤ 28.57 g	1						
28.58–42.86 g	0.064	0.117	0.299	0.585	1.066	0.847	1.342
42.87–71.43 g	0.27	0.113	5.737	0.017	1.31	1.05	1.634
≥ 71.44 g	0.128	0.111	1.324	0.25	1.137	0.914	1.414
Daily milk and milk products intake							
Q1(≤ 14.29 g)	1						
Q2(14.30–142.86 g)	−0.063	0.117	0.288	0.591	0.939	0.747	1.181
Q3(142.87–227.5 g)	0.052	0.121	0.186	0.666	1.053	0.831	1.335
Q4(≥ 227.51 g)	0.048	0.119	0.166	0.684	1.049	0.832	1.324
Daily eggs intake(ref: ≤ 1 egg)	−0.193	0.206	0.879	0.348	0.825	0.551	1.234

*ref* reference, *CVD* cardiovascular disease, *WC* waist circumference, *BMI* body mass index.

The findings from the analyses stratified by age and sex can be found in Table 6. In both male and female, the prevalence of dyslipidemia rises with advancing age, with a more noticeable age-related pattern observed in females.Hypertension and diabetes are associated with an increased risk of dyslipidemia in both male and female . CVD, stroke and smoke were associated with dyslipidemia in male while family history of CVD, family history of cancer, daily vegetables intakes and daily seafood intakes were associated with dyslipidemia in female. Hypertension, diabetes, obesity, and frequent smoking were related to dyslipidemia in participants younger than 60. gender, education level, hypertension, diabetes, CVD, frequent smoking, former smoking, family history of CVD and daily seafood intakes were associated with dyslipidemia in participants aged 60 and older.

**Table 6:** Analysis of factors associated with dyslipidemia stratified by age and gender.

Strata	Variables	B	Se	Wald *x*^2^	P	OR	Lower	Upper
Male	Age							
	18–29	1						
	30–39	–0.285	0.292	0.954	0.329	0.752	0.424	1.333
	40–49	0.428	0.288	2.207	0.137	1.534	0.872	2.699
	50–59	0.484	0.285	2.873	0.09	1.622	0.927	2.838
	60–69	0.419	0.281	2.222	0.136	1.52	0.876	2.636
	70 and above	0.755	0.294	6.583	0.01	2.127	1.195	3.785
	Hypertension(ref: yes)	–0.442	0.145	9.321	0.002	0.643	0.484	0.854
	Diabetes(ref: yes)	−0.507	0.166	9.362	0.002	0.603	0.436	0.834
	CVD(ref: yes)	−0.705	0.24	8.629	0.003	0.494	0.309	0.791
	Stroke(ref: yes)	0.569	0.272	4.372	0.037	1.767	1.036	3.012
	Smoke							
	Never	1						
	Frequent	0.668	0.164	16.602	< 0.001	1.951	1.415	2.691
	Occasional	0.166	0.272	0.371	0.542	1.18	0.693	2.011
	Former	0.533	0.196	7.368	0.007	1.703	1.16	2.502
Female	Age							
	18–29	1						
	30–39	0.524	0.345	2.312	0.128	1.689	0.86	3.318
	40–49	1.256	0.344	13.331	< 0.001	3.51	1.789	6.887
	50–59	1.95	0.344	32.176	< 0.001	7.03	3.584	13.791
	60–69	2.755	0.345	63.95	< 0.001	15.728	8.005	30.9
	70 and above	2.559	0.355	52.015	< 0.001	12.929	6.449	25.921
	Education							
	Primary school and below	1						
	Junior middle school	0.425	0.255	2.781	0.095	1.53	0.928	2.523
	Senior middle school	0.655	0.205	10.185	0.001	1.926	1.288	2.881
	Under graduate and above	1.068	0.259	16.957	< 0.001	2.91	1.75	4.837
	Hypertension(ref: yes)	−0.524	0.14	14.051	< 0.001	0.592	0.45	0.779
	Diabetes(ref: yes)	−0.479	0.171	7.895	0.005	0.619	0.443	0.865
	Family history of CVD(ref: yes)	−0.847	0.273	9.603	0.002	0.429	0.251	0.733
	Family history of cancer(ref: yes)	−0.613	0.275	4.968	0.026	0.542	0.316	0.929
	Daily vegetables intake							
	Q1(≤ 200 g)	1						
	Q2(201–250 g)	−0.243	0.156	2.419	0.12	0.784	0.578	1.065
	Q3(251–500 g)	−0.309	0.152	4.117	0.042	0.734	0.545	0.99
	Q4(≥ 501 g)	−0.469	0.321	2.138	0.144	0.626	0.334	1.173
	Daily seafood intakes							
	≤ 28.57 g	1						
	28.58–42.86 g	0.07	0.163	0.183	0.669	1.072	0.779	1.477
	42.87–71.43 g	0.351	0.159	4.865	0.027	1.421	1.04	1.942
	≥ 71.44 g	0.068	0.157	0.188	0.664	1.071	0.787	1.457
Age < 60	Hypertension(ref: yes)	−0.65	0.169	14.799	< 0.001	0.522	0.375	0.727
	Diabetes(ref: yes)	−0.809	0.262	9.547	0.002	0.445	0.267	0.744
	BMI							
	Underweight	1						
	Normal	0.354	0.333	1.132	0.287	1.425	0.742	2.735
	Overweight	0.399	0.357	1.253	0.263	1.491	0.741	3
	Obesity	1.084	0.436	6.188	0.013	2.956	1.258	6.942
	Smoke							
	Never	1						
	Frequent	0.602	0.216	7.778	0.005	1.826	1.196	2.787
	Occasional	−0.112	0.39	0.082	0.775	0.894	0.417	1.92
	Former	0.486	0.441	1.217	0.27	1.626	0.686	3.855
Age ≥ 60	Gender(ref: male)	0.568	0.15	14.379	< 0.001	1.764	1.316	2.366
	Education							
	Primary school and below	1						
	Junior middle school	0.584	0.218	7.162	0.007	1.793	1.169	2.749
	Senior middle school	0.694	0.18	14.841	< 0.001	2.002	1.406	2.849
	Under graduate and above	1.047	0.237	19.537	< 0.001	2.849	1.791	4.532
	Hypertension (ref: yes)	−0.505	0.12	17.677	< 0.001	0.604	0.477	0.764
	Diabetes (ref: yes)	−0.461	0.133	12.104	0.001	0.63	0.486	0.818
	CVD (ref: yes)	−0.415	0.17	0.597	0.015	0.66	0.473	0.921
	Smoke							
	Never	1						
	Frequent	0.414	0.201	4.24	0.039	1.513	1.02	2.245
	Occasional	0.246	0.337	0.532	0.466	1.278	0.661	2.473
	Former	0.499	0.211	5.587	0.018	1.647	1.089	2.491
	Family history of CVD(ref: yes)	−0.9	0.271	11.003	0.001	0.406	0.239	0.692
	Daily seafood intakes							
	≤ 28.57 g	1						
	28.58–42.86 g	0.017	0.161	0.012	0.915	1.017	0.743	1.394
	42.87–71.43 g	0.319	0.154	4.313	0.038	1.376	1.018	1.859
	≥ 71.44 g	0.181	0.148	1.495	0.221	1.199	0.896	1.604

## Discussion

The present study investigated the prevalence and risk factors of dyslipidemia among community adults in Shangcheng district. The findings revealed that the overall prevalence of dyslipidemia in this region was 35.96%. Chinese National Nutrition and Health Survey (CNNHS) conducted in 2002, providing appropriate comparison data for our study, revealed that the prevalence of dyslipidemia in present study was nearly double that of the national data (18.6%)^24^. The median level of TC, TG, HDL-C, and LDL-C were 4.78, 1.38, 1.38, and 2.64 mmol/L, exhibiting an increase when compared to the findings of the previous study^25^. A positive association between age and dyslipidemia has presented in both full-sample data analyses and gender-stratified analyses,which has also been observed in other city in China^26^. Previous research has reported that men have a higher risk of dyslipidemia than women^27^, which is contrary to the findings of our study. Our study found that women are more likely to be affected by dyslipidemia, especially those aged 60 years and above . This might be due to that menopause is a significant risk factor of dyslipidemia in older women. Study reveals that menopause leads to changes in lipid profile through reducing HDL-C, and elevating TC, TG and LDL-C^28^. The study showed positive association between education level with dyslipidemia prevalence ,which is in the line with previous study^29^, the possible explanation for this result is that participants with higher education levels may spend more time sitting in the office, have little time to exercise, frequently consume high fat foods, and suffer from a work-related mental health problem^30^. The present study also identified several common risk factors such as hypertension, diabetes, smoking and obesity in our study population, which is consistent with previous studies^31–34^. A family history of CVD and cancer may also lead to an increased risk of dyslipidemia, which has been confirmed in previous study^35,36^. It was interested that alcohol consumption was negatively associated with dyslipidemia, the possible reason may be that alcohol reduced the activity of cholesterol ester transformation from HDL to atheromatic molecules, subsequently increased the circulating levels of HDL-C^37^. However, this association was not observed in the analyses stratified by age and sex. Further studies are needed to validate this findings and to investigate the potential mechanisms. A negative association between dyslipidemia and stroke in male was found in present study, which is consistent with previous studies conducted on female^38,39^, however, the relationship between lipid level and stroke remains controversial and the mechanism has not been elucidated. Further gender specific research is needed.

Moderate intake of vegetables contributed to lower blood lipids, which is confirmed in other researches^40,41^. Daily seafood intakes is positive associated with dyslipidemia, which is inconsistent with other studies. numerous studies have shown that seafood especially fish contained abundance of n-3 polyunsaturated fatty acids (n-3 PUFAs: eicosapentaenoic acid [EPA] and docosahexaenoic acid [DHA]) in fish oils, such as sardine and saury fish^42^, These elements had diverse cardioprotective effects. The conflicting results may be attributed to the heterogeneity of study designs and/or source of dietary seafood. Another possible explanation could be that individuals with dyslipidemia who adhere to a self-reported diet exhibit a more favorable dietary intake compared to the broader population. The present study was unable to provide a detailed breakdown of various seafood categories, which represents a limitation of the study and suggests a potential avenue for future research.

Our study possesses several strengths. Firstly, instead of employing principal component or factor analysis to examine the relationship between diet and blood lipid, we directly examined each food group, allowing for a more nuanced understanding of the role of each food item. Secondly, the sample of this study was obtained using a multistage random sampling technique to ensure the representative of the population in this region. The research was advantageous for the subjects as it involved a thorough medical assessment. Furthermore, the utilization of a standardized training manual to instruct the research aides facilitated a uniform and methodical approach to data gathering, thereby enhancing the reliability of the measurements. Finally, the abundance of health data, coupled with advanced statistical methods, presents an opportunity to uncover previously unrecognized relationships that were not apparent using traditional statistical methods and smaller datasets. This has the potential to enhance our understanding of the contributing factors of dyslipidemia and provide a more comprehensive insight into the condition. However, our study has several limitations. The inherent features of the cross-sectional design of the study limit any potential inference of causality and temporal relationship between the variables, despite rigorous training and questionnaire quality control of our surveyors, some self-reported items were inevitably subject to recall bias, such as smoke, drink. Additionally, the number of case samples did not allow for a detailed analysis of the different types of dyslipidemia, and some dietary categories could not be further refined. Future research with a larger sample size is needed to explore those factors associated with different types of dyslipidemia.

In summary, the prevalence of dyslipidemia remains high among population aged 18 years and above in this region. Risk factors such as gender, age, education, hypertension, diabetes, cardiovascular disease, stroke, obesity, smoking, drinking, family history of cardiovascular disease, family history of cancer, daily vegetables intakes, daily seafood intakes related to dyslipidemia and varied across age and gender groups.

## Conclusion

According to our study, the overall prevalence of dyslipidemia among individuals aged 18 years and above in Shangcheng district remains high. Multiple factors are associated with dyslipidemia, emphasizing the need for heightened awareness and efficient measures to be adopted by local healthcare providers in order to reduce the prevalence of dyslipidemia among adults.

## Data Availability

Raw clinical data is not permitted for sharing according to the terms of the informed consent. The summary information or other non-subject level analyses for the datasets used and/or analyzed during the current study available from the corresponding author on reasonable request.
